# Exploring effects of gut microbiota on tertiary lymphoid structure formation for tumor immunotherapy

**DOI:** 10.3389/fimmu.2024.1518779

**Published:** 2025-03-07

**Authors:** Yuqing Liu, Fan Li, Juanjuan Wang, Rongcun Yang

**Affiliations:** ^1^ Department of Immunology, Nankai University School of Medicine, Nankai University, Tianjin, China; ^2^ State Key Laboratory of Medicinal Chemical Biology, Nankai University, Tianjin, China; ^3^ Translational Medicine Institute, Affiliated Tianjin Union Medical Center of Nankai University, Nankai University, Tianjin, China

**Keywords:** gut microbiota, tertiary lymphoid structure, immunotherapy, Tregs, tumor

## Abstract

Anti-tumor immunity, including innate and adaptive immunity is critical in inhibiting tumorigenesis and development of tumor. The adaptive immunity needs specific lymph organs such as tertiary lymphoid structures (TLSs), which are highly correlated with improved survival outcomes in many cancers. In recent years, with increasing attention on the TLS in tumor microenvironment, TLSs have emerged as a novel target for anti-tumor therapy. Excitingly, studies have shown the contribution of TLSs to the adaptive immune responses. However, it is unclear how TLSs to form and how to more effectively defense against tumor through TLS formation. Recent studies have shown that the inflammation plays a critical role in TLS formation. Interestingly, studies have also found that gut microbiota can regulate the occurrence and development of inflammation. Therefore, we here summarize the potential effects of gut microbiota- mediated inflammation or immunosuppression on the TLS formation in tumor environments. Meanwhile, this review also explores how to manipulate mature TLS formation through regulating gut microbiota/metabolites or gut microbiota associated signal pathways for anti-tumor immunity, which potentially lead to a next-generation cancer immunotherapy.

## 1 Introduction

Anti-tumor immunity, including innate and adaptive immune responses is critical in inhibiting tumorigenesis and tumor development. Notably, anti-tumor adaptive immunity needs specific lymph organs such as lymph nodes (secondary lymphoid organs) and tertiary lymphoid structures (TLSs), also known as ectopic lymphoid structures (ELSs) or tertiary lymphoid organs (TLOs) (1). When TLSs are located in close vicinity of tumor, they are active sites of inducing adaptive immune responses against tumor (2, 3). Indeed, emerging studies have already revealed the contribution of the TLSs to adaptive immune response against tumor (4) such as the production of antibodies that can mark tumor cells for complement-mediated lysis, antibody-dependent cellular cytotoxicity or opsonization (5). Increased activation markers has also been observed on the T cells in TLSs, as compared with other tumor-resident T cells in melanoma (6). These TLSs were highly correlated with improved survival outcomes in many tumors, such as breast cancer, hepatocellular cancer (HCC), colorectal cancer (CRC), melanoma, gastric cancer, head and neck squamous cell cancer (HNSCC), lung cancer and sarcoma (7, 8). However, TLS formation remains to be further clear.

**Figure 1:**
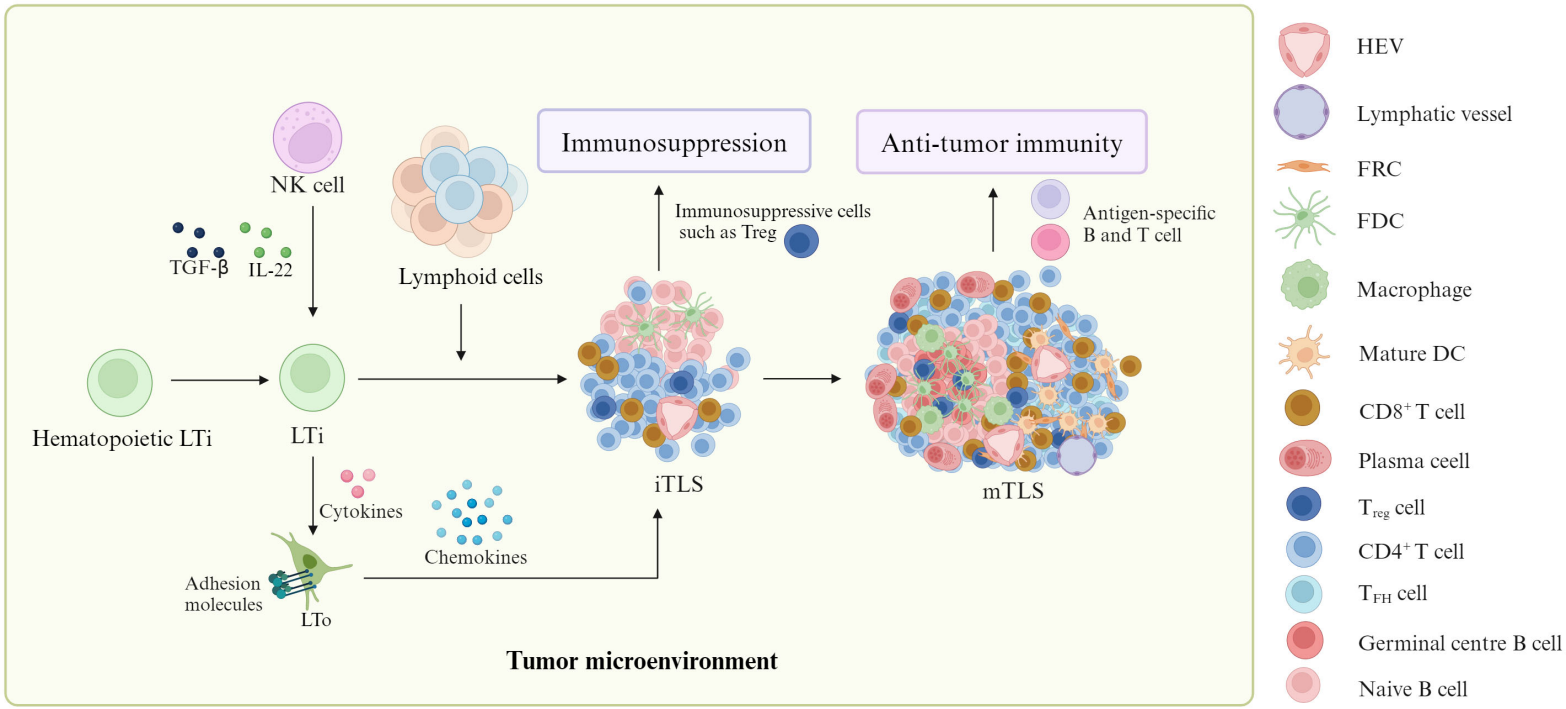
Formation of TLS. In tumor environment, lymphoid tissue inducers (LTi) can produce cytokines to work on lymphoid tissue organizers (LTo). These LTo express adhesion molecules and chemokines to recruit immune cells from adjacent high endothelial venules (HEV) for mature tertiary lymphoid structure (mTLS) formation through immature TLS (iTLS) containing a lot of immunosuppressive cells such as T regulatory cells (Tregs). MTLSs can produce antigen-specific T and B cells to promote anti-tumor immunity. Notably, natural killer (NK) cells could be converted into LTi. ITLS can cause immunosuppression through immunosuppressive cells such as Treg.

Although the factor(s) and mechanism(s) influencing TLS formation are incompletely clear, some studies have shown that TLSs are generally derived from hematopoietic LTi (lymphoid tissue inducers), which can interact with stromal cells known as LTo (lymphoid tissue organizers) to form mature TLS through immature TLS (**Figure 1**) (2, 8, 9). The stromal cells can express adhesion molecules and chemokines to recruit immune cells from adjacent high endothelial venules (HEV) for TLS formation. Notably, LTi is also converted from nature killer (NK) cells under the stimulation of transforming growth factor (TGF)-β and IL-12 (10–13). In addition, chronic inflammation also plays a critical role in TLS formation (14). It not only induces immune cells such as T helper cells secreting IL-17 (T_H_17) to become LTi but also produces widely cytokines and chemokines, which are necessary to TLS formation (14). The requirement of chronic inflammation for TLS formation not only emerges from cancer mouse models but also from clinical observations (15–17).

Gut microbiota can regulate the occurrence and development of inflammation, which potentially affect TLS formation. Indeed, the gut microbes, which appear in tumor tissue can promote TLS formation. For example, *Helicobacter* pylori (*H. pylori*) inside tumors could promotes the formation of TLS to trigger anti-tumor immune responses (18). The gut microbiota *Lachnoclostridium* was also related to the existence of intratumoral TLSs in hepatocellular carcinoma (19). Besides the microbes from gut microbiota in tumor tissues, the metabolites derived from gut microbiota also potentially affect the TLS formation. These metabolites can enter tumor tissues through bloodstream. Since gut microbiota and its metabolites (microbiota/metabolites) are one of the major environmental factors that affects TLS formation (20), we here summarize the potential contribution of gut microbiota/metabolites mediated inflammation or immunosuppression on the TLS formation in tumor environments, especially on the different immune cells such as macrophages (Macs), dendritic cells (DCs), Tregs (regulatory cells), LTi, Tfh (follicular helper T cell) and B cell in TLSs (21). Since there exists the association of TLS with a favorable response to immunotherapy against tumor, studies are employing different approaches to induce TLS formation *in vivo*. This review also explores the potential opportunity to promote TLS formation for defensing against tumor through regulating gut microbiota/metabolites or their signal pathway(s) in immune cells. Notably, the effects of gut microbiota/metabolites on TLS formation have only just begun. Of course, besides gut microbiota, others such as tumor-derived factors also influence TLS formation.

## 2 TLSs in tumor environments

TLSs, as ectopic lymphoid organs develop in non-lymphoid tissues at the sites of chronic inflammation such as tumors (22). They share the vasculature, chemokines, cellular compartments, spatial organization, and function with secondary lymphoid organs, especially lymph nodes, where adaptive anti-tumor cellular and humoral responses can be generated (23).

TLSs in cancer tissues are heterogeneous (7). Their cell composition and distribution is also different in different cancer types (24), ranging from disorganized cellular aggregates such as early TLS or immature (iTLS) to well-organized and structured organs which are similar to secondary lymphoid organs (SLOs) such as lymphoid nodes (2, 25). Thus, TLSs include multiple lymphoid structures, from immune cell aggregates to organized structures, which form primary follicle (PFL) or secondary follicle (SFL) with a germinal center (GC) in the tumor microenvironment (TME) (2).

### 2.1 Lymphoid aggregate and immature TLS

Lymphoid aggregates are composed of a few B cells and T cells without any follicular dendritic cells (FDCs) (23). In immature TLS (iTLS), besides T, B cells and FDCs, there often are aggregates of immune suppressive cells that suppress anti-tumor immunity such as higher programmed death-ligand 1 tumor-associated Macs (26), immature DCs (26), regulatory T cells (26, 27), PD-1^high^ CD8 T cells (26, 28) and PD-1^high^CD4 T cells (28). Typically, a low CD8/Treg cell ratio in lung squamous cell carcinoma (LUSC) (29), interleukin (IL)-10 expressing monocytes and T cells in pancreatic ductal adenocarcinoma (PDAC) (30) and phosphoprotein 1 (SPP1)^+^ Macs in the lung cancer activation modules (LCAMs) (31) have also been reported. Notably, one study also exhibited that there were only minor differences in immune cell frequencies in immature versus mature TLS with the exception of B cells (28).

### 2.2 Mature TLS

Signatures of mature TLSs have been described in other papers (2, 23). They have typical structures comprised of an internal B-cell zone surrounded by T cell-rich area, which includes CD8 cytotoxic T, CD4 Th-1 and Tfh lymphocytes, as well as LAMP3^+^ DCs (32). The B cell zone has a network of follicular DCs (FDCs), which express CD21 in PFL as well as CD23 in SFL. These TLSs reveal clear active GCs within the B-cell area with the mature DCs (33), antigen-experienced CD4, CD8 and B cells (34).

### 2.3 Inflammation and TLS formation

**Figure 2:**
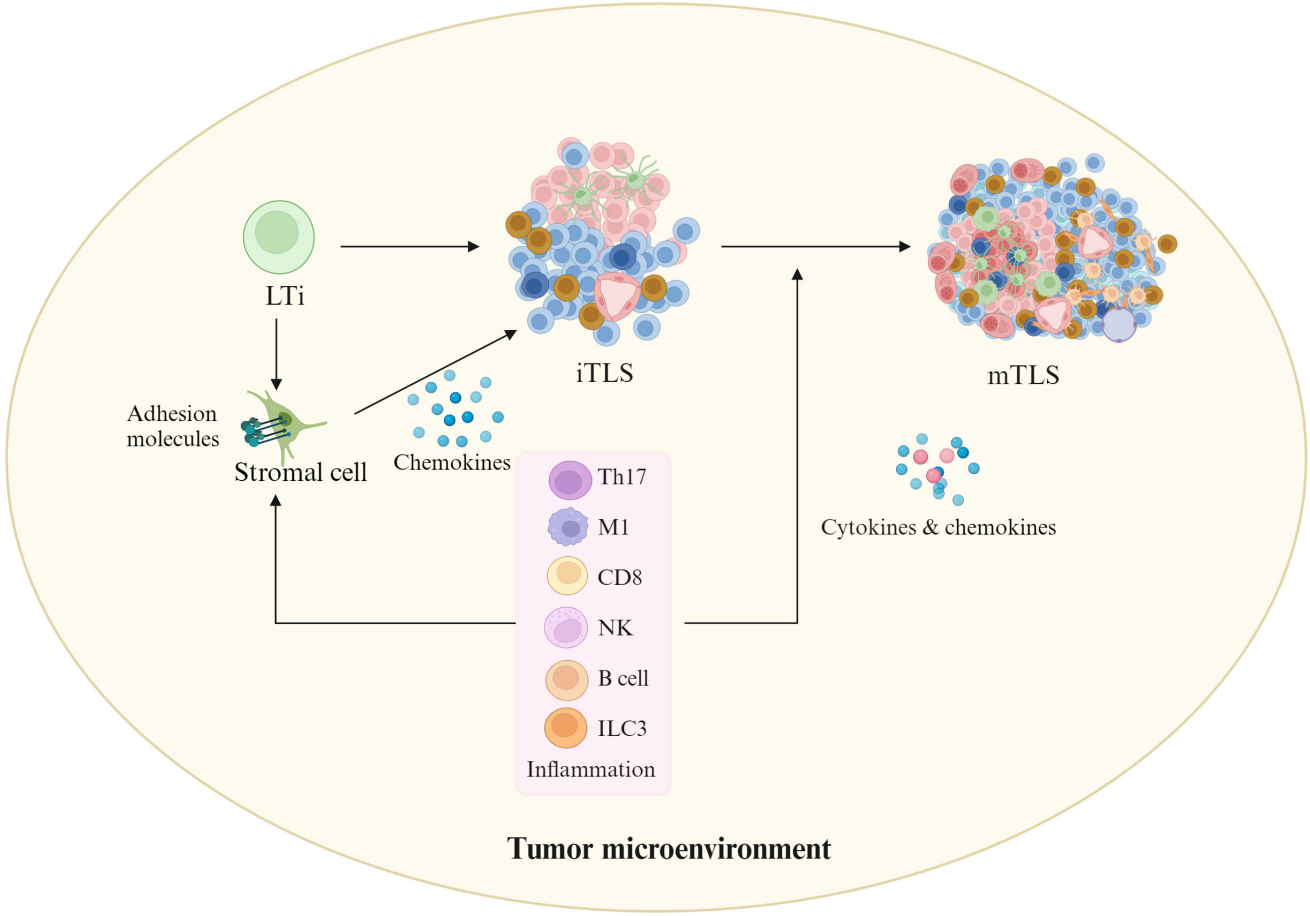
Effects of inflammation on TLS formation. In tumor environments, inflammatory cells such as ILC3 (innate lymphoid cell 3), T helper cells secreting IL-17 (Th17), M1-polarized Macs, effector CD8 T cells and natural killer (NK) cells, as well as B lymphocytes may replace LTi cells. They can interact with stromal cells to initiate TLS formation in the absence of LTi cells. In addition, inflammation also promotes immature TLSs into mature TLSs through cytokines and chemokines. Cells in iTLS and mTLS, see **Figure 1**.

Inflammation plays a critical role in TLS formation (**Figure 2**). TLS formation relies on the cytokine signaling network between heterogeneous cell populations such as lymphocytes, stromal cells and cancer cells (35). TLSs can be generally derived from LTi, which can produce cytokines such as lymphotoxin α1β2 (LTα1β2), TNFα and IL-17 (8, 36), which interact with the receptors on the LTo cells such as stromal cells. These LTo cells express adhesion molecules such as VCAM1, ICAM1, and MADCAM1, and chemokines such as CXCL13, CXCL12, CCL21 and CCL19, which can recruit immune cells from adjacent HEV to participate in TLS formation. Interestingly, HEV can express PNAD (peripheral lymph node addressin), a L-selectin receptor, which is necessary for leukocytes to the TLSs. However, in response to chronic inflammatory, several immune cell populations can potentially replace LTi cells, including T_H_17 (37, 38), natural cytotoxicity receptor (NCR)^+^ ILC3 (NCR^+^ILC3) (39), M1-polarized Macs (40), effector CD8 T cells and NK cells (41), as well as B lymphocytes (42). These cells can interact with stromal cells to induce TLS formation in the absence of LTi cells. Some local stromal cells such as fibroblasts can be acted as LTo (43). In addition, inflammation also promotes immature TLSs into mature TLSs through cytokines and chemokines (23) such as interleukin-17A (IL-17A), which has been shown to be involved in the formation of TLSs (44). Indeed, the requirement of chronic inflammation for TLS formation not only emerges from cancer mouse models but also from clinical observations (45, 46).

## 3 Gut microbiota metabolites and their receptors

Gut microbiota/metabolites play a critical role in the development of LTO. Since TLSs are similar to secondary lymphoid organs such as LN (47, 48), it may be useful to predict the effects of gut microbiota/metabolites on the TLS formation. Indeed, the microbiota has been shown to promote formation of TLSs (23). For instance, *H. hepaticus* promoted enrichment of TLSs in a mouse model of colorectal cancer (CRC) (18). Notably, although the effects of gut microbiota/metabolites on the differentiation and function of immune cells have been widely studies, the role of gut microbiota/metabolites in TLS formation has only just begun.

Gut microbiota/metabolites not only affect inflammatory immune cells but also immunosuppressive cells, which can potentially affect formation of TLSs, typically metabolites derived from gut microbiota such as short chain fatty acids (SCFAs) (49, 50), bile acid (BA) (50–52) and tryptophan (Trp) metabolites (53, 54), and other metabolites, which have been reviewed by us and others. SCFAs such as butyrate, propionate, and acetate are from dietary fiber fermentation by gut microbiota in the cecum and colon (49, 50). The primary BAs cholic acid (CA) and chenodeoxycholic acid (CDCA) generated in the liver can conjugate CDCA, DCA or CA to one or more amino acids by bacteria (55). Four distinct ways including deconjugation, oxidation, dehydroxylation and epimerization in human are used to transform BAs (56). While BAs are deconjugated, BAs can be converted into secondary BAs deoxycholic acid (DCA) and lithocholic acid (LCA) through bacterial bile acid hydrolases (57) and dehydroxylases (58) to remove 7α or 7β-hydroxyl groups from primary BAs in the colon. In addition, a range of oxo-, iso- and epi-derivatives such as 3-oxoLCA, 7-oxoCDCA, 12-oxoCA, 7-oxoCA, 12-oxo-DCA, 3-oxo-LCA, 3-oxo-allo-LCA, iso-LCA, iso-alloLCA, allo-LCA and 3-ketoLCA are also generated by gut bacteria (56, 59, 60). Gut microbiota also metabolizes Trp into tryptamine and indole derivatives, such as indole, indole-3-acid-acetic (IAA), indole acetic acid, indole-3-propionic acid (IPA), indole-acrylic acid (IA), indole-3-aldehyde (IAld), indole-3-pyruvate (IPy), indole-3-acetamide (IAM), indoxyl sulfate and tryptamine (61, 62). In addition, some bacteria also produce kynurenine (Kyn) and downstream metabolites such as 3-hydroxyanthranilic acid (3-HAA) through enzymes homologous to those of the eukaryotic kyn pathway (63).

These gut microbial metabolites can regulate the differentiation and function of immune cells through various receptors. SCFAs are through G-protein coupled receptor (GPR)41 (also known as free fatty acid receptor (FFAR3)), GPR109a (also called hydroxycarboxylic acid receptor 2 (HCA2)) and GPR43; Whereas cellular membrane receptors such as G-protein BA receptor 1 (GPBAR1) known as Takeda G protein-coupled receptor 5 (TGR5), and nuclear receptors such as farnesoid X receptor (FXR), pregnane X receptor (PXR), vitamin D receptor (VDR), liver-X-receptor (LXR), constitutive androstane receptor (CAR), and retinoid related orphan receptor (RORγt) (64), are used by BA derivatives. A variety of Trp-indole metabolites are also through receptors such as aryl hydrocarborn receptor (AhR) and PXR to exert their functions (65).

## 4 Development of lymphoid tissue organs depends on gut microbiota

The gut microbiota is a complex ecosystem of approximately 100 trillion microorganisms inhabiting the human gut, including bacteria, viruses, fungi and protozoa (66). The vast majority is represented by *Firmicutes* (gram-positive, 60–80%) and *Bacteroidetes* (gram-negative, 20–30%) along with *Proteobacteria* and *Actinobacteria* (67), which can induce either inflammation or immunosuppression in the individuals. The gut microbiota plays a critical role in the development of lymph organs, including primary lymphoid organs such as thymus (68) and bone marrow cells (69), and second lymphoid organs such as lymph node (LN) and Peyer’s patches (70), spleen, tonsils and mucosa-associated lymphoid tissues, which support immune surveillance in the mammalian organisms. Indeed, studies have found that secondary lymphoid organs of germ-free mice are under-developed (71). Antibiotic treatment, which resulted in decreased gut microbiota, also reduced DC and effector CD8 T cell responses, and attenuated responses to immune checkpoint blockade therapy (72).

## 5 Promotion of gut microbiota/metabolites on mature TLS formation

**Figure 3:**
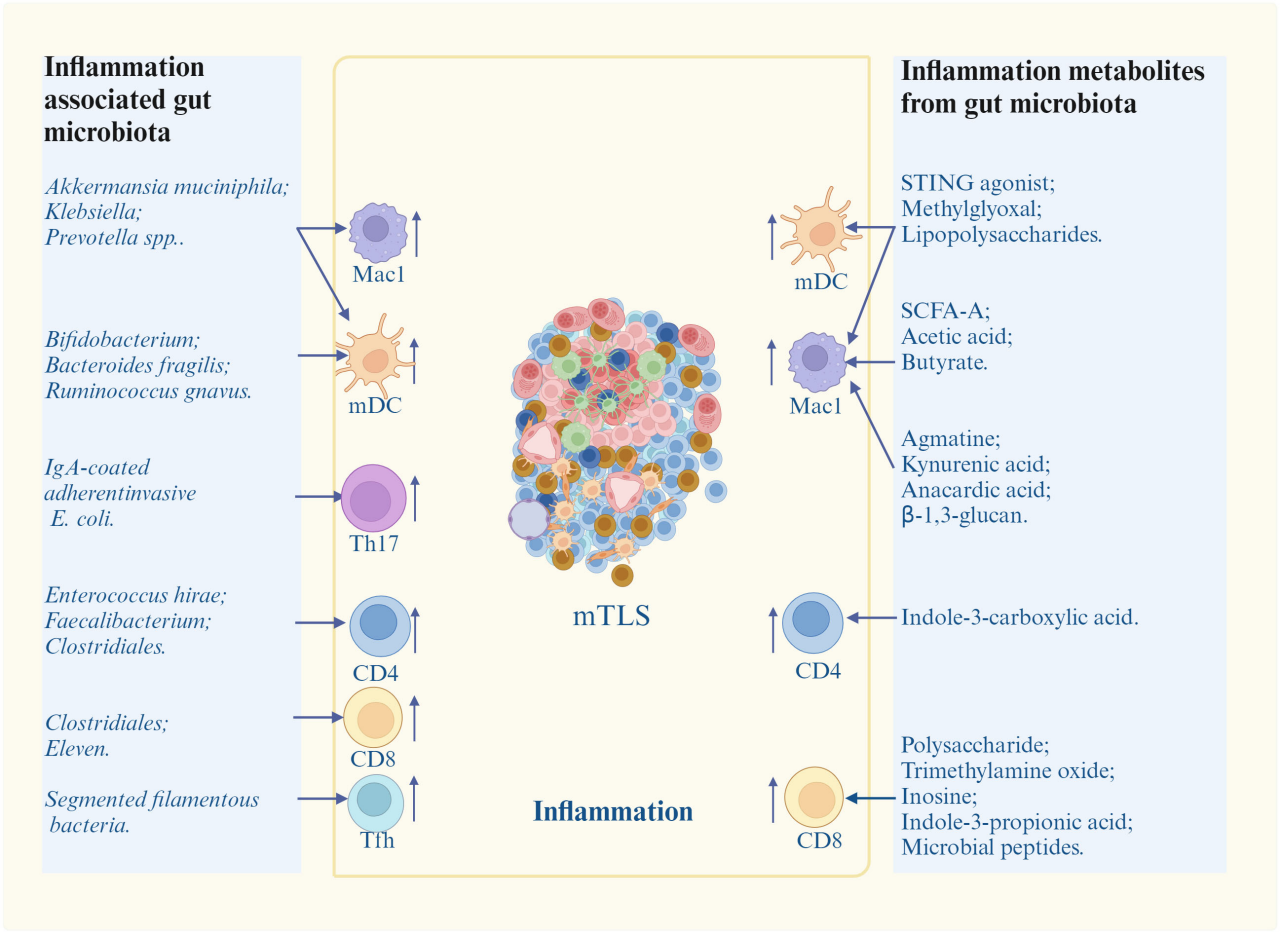
Promotion of inflammation-associated gut microbiota/metabolites on mature TLS (mTLS) formation. Inflammation associated gut microbiota/metabolites induce and/or promote mature TLS formation through different inflammatory immune cells. mDC, nature dendritic cells; Mac1, inflammatory macrophages; Th17, T helper 17 cells; Tfh, T follicle helper cells; STING, stimulator of interferon genes; SCFA-A, short chain fatty acid A. Cells in mTLS, see **Figure 1**.

Since inflammation plays a critical role in TLS formation, inflammation-associated gut microbiota/metabolites can potentially promote TLS formation (**Figure 3**).

### 5.1 Gut microbiota and structural components

Multiple bacteria can cause inflammation such as *Akkermansia muciniphila* (*A. muciniphila*) (73, 74), *Prevotella* spp (75), *Klebsiella* (76), *Bifidobacterium* (77, 78), *Bacteroides fragilis* (*B. fragilis*) (79), *B. fragilis* (80), *Ruminococcus gnavus* (81), *Segmented filamentous bacteria (SFB)* (82), *IgA-coated adherent-invasive* E. coli *(AIEC)* (83), *Clostridiales* (84), *Faecalibacterium* (85), *Enterococcus hirae* (86, 87) and *Eleven* strains (79). *A. muciniphila* could produce cdAMP to activate stimulator of interferon genes (STING)-interferon pathways of immune cells such as DCs and Macs to produce cytokines (73, 74). *Prevotella* spp up-regulated pro-inflammatory responses in Macs (75). *Klebsiella* activated NF-κB and promoted the secretion of pro-inflammatory IL-1, IL-6 and TNF-α in Macs (76). *Bifidobacterium* could alter the functional capacity of DCs to induce CD8 T cell proliferation and Th1 differentiation, and also IFNγ production (77, 78); *Bacteroides fragilis* (*B. fragilis*) could stimulate DC maturation to induce IL-12-dependent Th1 cell immune responses (79). *B. fragilis* also induced Mac polarization to M1 (80). *Ruminococcus gnavus* induced TNF-α secretion of DCs through pro-inflammatory polysaccharide (PSA) (81). *Segmented filamentous bacteria (SFB)* induced differentiation of Tfh cells in Peyer’s patches thorough DC-mediated inhibition on IL-2-related pathway, and also directed Tfh trafficking to lymphoid tissues responsible for antibody production (82). IgA-coated adherent-invasive *E. coli (AIEC)* triggered Th17 cells activation to increase systemic immune responses (83); *Clostridiales* promoted antigen presentation to promote CD4 and CD8 T cell function in melanoma patients (84). *Faecalibacterium* increased CD4 T cell proportion in peripheral blood in metastatic melanoma patients (85). *Enterococcus hirae* could induce the polarization of immune cells towards a Th1 phenotype in secondary lymphoid organs in P815 mastocytomas established in syngenic DBA2 mice (86, 87). *Eleven* strains robustly induced IFN γ^+^CD8 T cells in syngeneic tumor models (79). Bacteria *C. rodentium* induced pro-inflammatory Th17 cells (88).

Bacterial structural components also modulate immune cells through different receptors such as Toll-like receptors (TLR) and nod-like receptors (NLR). There have existed considerable literatures on various TLR and NLR ligands from gut microbiota impacting immune cells. For example, lipopolysaccharides (LPSs), a component of the Gram-negative bacterial outer membrane, was identified as a key contributing factor in the initiation and progression of inflammation (89). It could promote production of pro-inflammatory cytokines (TNF, IL-17, IL-22, etc.) in immune cells such as monocytes, Macs, and Kupffer cells, which expressed LPS receptors such as Toll-like receptor 4 (TLR4) (90). Bacterial-derived lipoteichoic acid could exert strong effects on immune cells via host receptors and targeted molecules. Microbial peptides also activated tumor-infiltrating lymphocytes in the tumor such as glioblastoma (91).

In conclusion, since the gut microbiota and its structural components can promote inflammation through stimulating different immune cells, they should be effective in promoting TLS formation against tumors.

### 5.2 Gut microbiota derived metabolites

**Figure 4:**
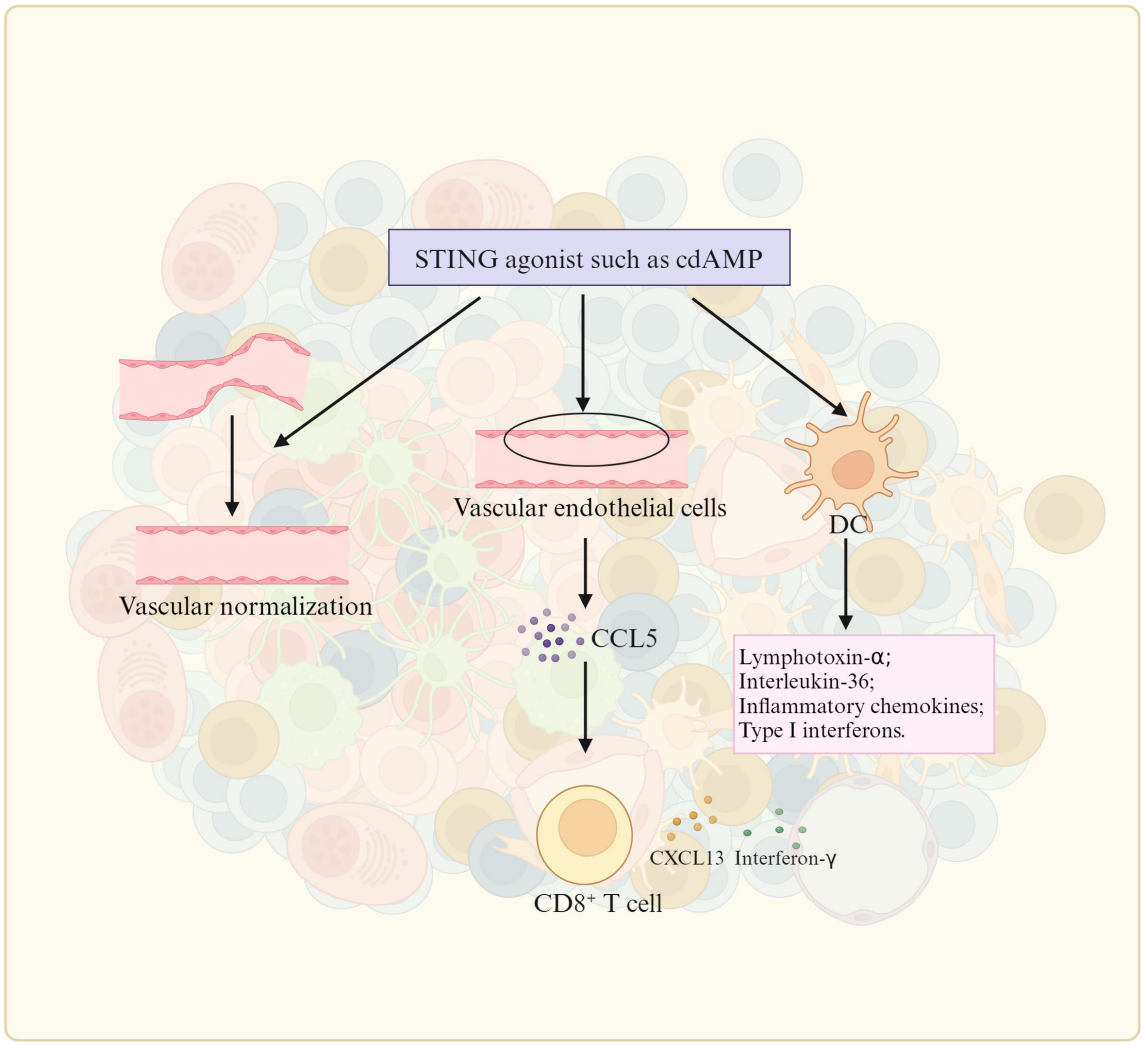
STING agonist promotes TLS formation. The stimulator of interferon genes (STING) promote TLS formation by upregulating the expression of TLS-promoting factors such as lymphotoxin-alpha (LTa), IL-36, type I IFN, inflammatory chemokines by DC (dendritic cells), vascular normalization, and CCL5 (CC chemokine ligand-5) production in vascular endothelial cells.

There are a lot of gut microbiota derived metabolites, which can cause inflammation such as stimulator of interferon gene (STING) agonists (92), methylglyoxal (93), SCFAs (94), agmatine (95), kynurenic acid (KYNA) (96), anacardic acid (97), tryptophan (Trp) metabolites (98), trimethylamine oxide (TMA) (99), inosine, microbial peptides (91) and β-1, 3 –glucan (100). STING agonists can promote TLS formation (**Figure 4**). Chelvanambi et al. demonstrated that intratumoral administration of STING agonist ADU-S100 could cause sustained inflammation in the TME of B16 melanomas, and production of cytokines/chemokines (LTα, LTβ, CCL19 and CCL21, as well as LIGHT), which could promote TLS formation (92). CD11c^+^ DCs activated by STING agonist ADU S-100 also upregulated expression of lymphotoxin-α (LTA), IL-36, chemokines and type I interferons *in vitro* and *in vivo*, which could promote TLS formation (92). The cyclic guanosine monophosphate–adenosine monophosphate synthase (cGAS)-STING pathway also initiated TLS formation through chemokine mediated cross-talk between endothelial cells and T cells (101). Mechanically, cell-intrinsic cGAS could lead to CCL5 production in vascular endothelial cells. Then, peripheral CD8 T cells were recruited to produce CXCL13 and interferon-γ to promote TLS formation (101). Methylglyoxal from gut microbes could boost radioimmunotherapy in rectal cancer by triggering endoplasmic reticulum stress and cGAS-STING activation, which could induce TLS formation (93). There existed a positive correlation between anti-PD-1/PD-L1 responses and bacteria that produce SCFAs like *Eubacterium*, *Lactobacillus*, and *Streptococcus* (102). SCFA-A, a metabolite of gut microbiota was also involved in the activation of T cells and induction of M1 Macs, thereby enhancing the anti-tumor effects of anti- PD-1 antibody therapy (103). *B. thetaiotaomicron*-derived acetic acid had the potential to modulate the polarization of pro-inflammatory Macs, which promoted CD8 T cell function in hepatocellular carcinoma (104). Gut microbiota-derived butyrate inhibited the immunosuppressive factors PD-L1 and IL-10 in tumor-associated Macs in gastric cancer (94). Butyrate also promoted the production of antitumor cytokines in cytotoxic CD8 T cells by regulating T-cell receptor (TCR) signaling pathway in patients with non-small cell lung cancer (NSCLC) (105). Gut microbiota-derived metabolite agmatine could suppress the Rnf128-mediated ubiquitination of β-catenin to upregulate the genes, which could activate Wnt pathway, including Cyclin D1, Lgr5, CD44 and C-myc. The activated Wnt signaling pathway could upregulate pro-inflammatory cytokines (IL-6 and TNF-α) and downregulate anti-inflammatory cytokine (IL-10) in colorectal tumorigenesis (95). Gut microbiota-induced kynurenic acid induced GPR35-positive Macs to promote inflammation (96). Anacardic acid could also activate Macs thorough mitogen-activated protein kinases (MAPKs) (97). Anacardic acid promoted tumor-infiltrating immune cells such as tumor-infiltrated NK cells and CTLs through inducing production of a neutrophil extracellular trap in breast cancer models (97). *L. gallinarum*-derived Trp metabolite indole-3-carboxylic acid (ICA) could compete with Kyn for binding site on AhR on CD4 T cells to inhibit Treg differentiation *in vitro* (98). I3AA, a Trp metabolite made by a gut microbia induced pro-inflammatory T cells, and reduced Treg subset *in vivo* and iTreg development *in vitro* via regulating response to TGFβ (106). IPA derived from *L. johnsonii*, could enhance the differentiation of CD8 T cells through H3K27 acetylation at the super-enhancer (SE) of Tcf7 gene in pan-cancer (107). In mice and humans, PSA generated by *L. delbrueckii* could induce CCR6^+^CD8 T cells (108). The choline or carnitine in foods were metabolized to generate trimethylamine (TMA) by the gut microbiota, which enters the liver through portal vein. Then TAM, which was catalyzed to produce trimethylamine oxide (TMAO) in liver, was demonstrated to promote CD8 T cell-mediated anti-tumor immunity in mouse models of triple-negative breast cancer (99). Inosine, a purine metabolite of *A. muciniphila* and *B. pseudolongum*, strengthened differentiation and proliferation of T cells (109), induced differentiation of B cells, and antibody production via activating Macs (109), and promoted antitumor immunity through Th1 differentiation and effector function of T cells (77). Some bacterial species, which could produce inosine or its metabolite hypoxanthine, induced T-helper 1 differentiation and effector functions via inosine-A2AR-cAMP-PKA pathway (110). The systemic administration of β-1,3-glucan from a fungal element promoted cytokine secretion (100). Thus, metabolites derived from gut microbiota, which can induce inflammation, also potentially result in mature TLS formation.

## 6 Suppression of gut microbiota/metabolites on mature TLS formation

**Figure 5:**
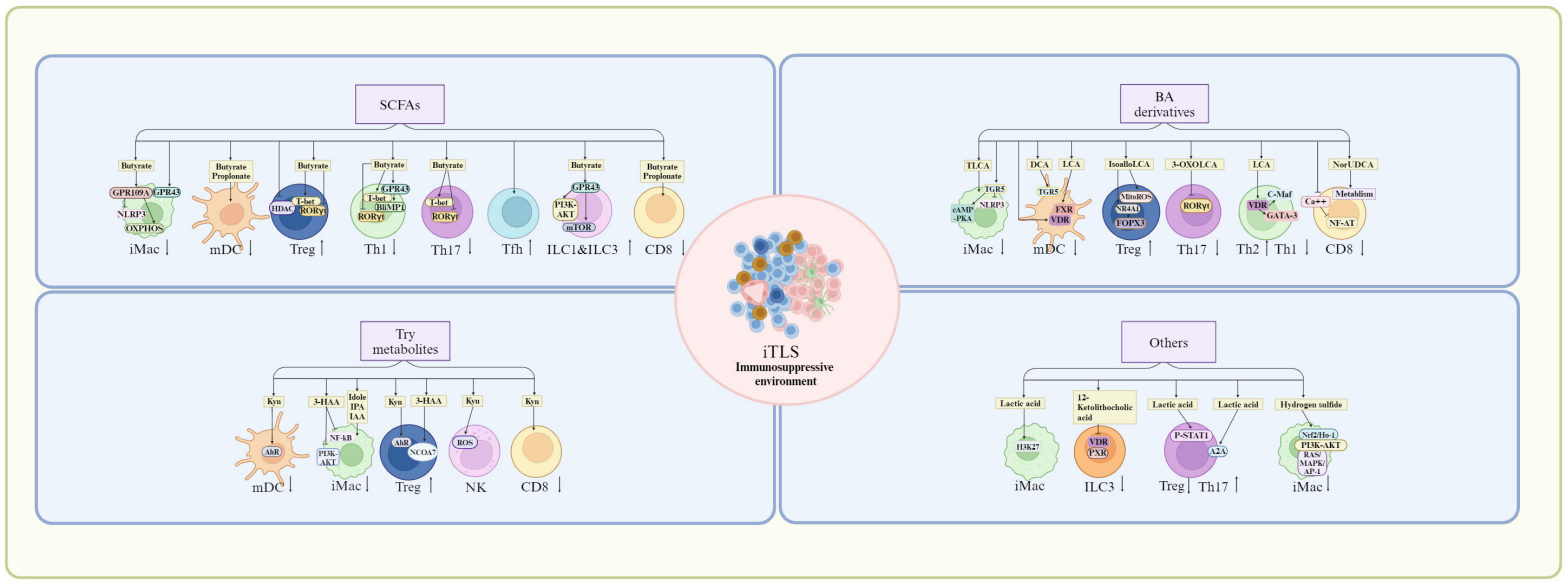
Suppression of anti-inflammation gut microbiota/metabolites on TLS formation. Anti-inflammation gut microbiota/metabolites can potentially inhibit the TLS formation by immunosuppressive cells or immature immune cells through their receptors such as short chain fatty acid (SCFA) receptors such as G-protein coupled receptor (GPR)43 and GPR109, bile acid (BA) derivative receptors such as farnesoid X receptor (FXR), VDR (Vitamin D receptor), retinoid related orphan receptor (RORγt), and tryptophan-indole metabolites receptors such as aryl hydrocarborn receptor (AhR). mDC, mature dendritic cells; iMac, inflammatory macrophages; Th17, T helper 17 cells; Tfh, T follicle helper cells; Treg, T regulatory cells; ILC, innate lymphoid cells; NLRP3, nucleotide-binding oligomerization domain (NOD)-like receptor family pyrin domain-containing 3; OXPHOS, oxidative phosphorylation; HDAC, histone deacetylase; PI3K, phosphoinositol-3 kinase; AKT, protein kinase B; mTOR, mammalian target of rapamycin; NF-κB, nuclear factor kappa-light-chain-enhancer of activated B cells; NR4A1, nuclear receptor subfamily 4 group A member 1; cAMP-PKA, cyclic adenosine monophosphate/protein kinase A; NCOA7, nuclear receptor co-activator protein 7; Nrf/Ho-2, nuclear factor E2-related factor/heme-oxygenase-2; A2A, A2A adenosine receptor; ROS, reactive oxygen species; RAS/MAPK, rat Sarcoma mitogen-activated protein kinase; AP-1, activator protein 1; ERK, extracellular-signal regulated kinase; JAK/STAT, Janus Kinase/Signal transducer of activated T-cells; TLCA, taurolithocholic acids; DCA, deoxycholic acid; LCA, lithocholic acid; 3-HAA, 3-hydroxyanthranilic acid; IPA, 3-indolepropionic acid; IAA, indole-3-acetic acid; Kyn, kynurenine. Cells in iTLS, see **Figure 1**.

Besides stromal cell and HEVs, mature TLSs are mainly composed of the different kinds of immune cells, including Macs, DCs, T cells such as (CD8, Tfh, Th1, Th17, and Treg) and B cells; Whereas in iTLS, besides T and B cells, there often are aggregates of immature and immune suppressive cells such as immunosuppressive Macs, immature DCs and Treg (26, 27), which can potentially inhibit mature TLS formation. Indeed, in a mouse LUAD model, Treg cell depletion not only enlarged the lung area covered by TLS but also increased levels of T cell proliferation and co-stimulatory ligand expression by DCs in tumor-associated TLSs (111). Repressing immunosuppressive cells such as anti-inflammatory macrophage or Breg cells also favored formation of TLS. Notably, the immunosuppressive cells can be regulated by gut microbiota/metabolites. Here, we mainly discuss the effects of anti-inflammatory gut microbiota or gut microbiota derived metabolites such as SCFAs, BA and Trp metabolites on these immunosuppressive cells, which can potentially inhibit the TLS formation (**Figure 5**).

### 6.1 Gut microbiota and structural components

Some bacteria from gut microbiota are resistant to inflammation such as *Bacteroides* (112, 113)*, Faecalibacterium prausnitzii* (112, 113), *Parabacteroides distasonis* (114), *Segmented filamentous bacteria* (90) and *Bacteroides* genus (115). *Bacteroides*, and *Faecalibacterium prausnitzii c*ould inhibit NF-κB to reduce pro-inflammatory cytokines such as IL-8 and TNF-α (112, 113). *Parabacteroides distasonis* inhibited the expression of pro-inflammatory cytokines (IL-1β, IL-6, IL-17A, and TNF-α), increased anti-inflammatory cytokines (IL-10), reversed Th17/Treg imbalance in the mesenteric LN (MLN) of mice with arthritis, and even induced M2-type polarization of Macs (114). *Segmented filamentous bacteria* was involved in the promotion of maturation of Treg cells, and also performed a mutualistic interaction with the gut microbiota by anti-inflammatory cytokines, such as IL-10 and TGF-β (90).

Interaction between segmented filamentous bacteria (SFB) and epithelial cells promoted formation of endocytic vesicles containing bacterial cell wall proteins, causing generation of non-inflammatory helper T (Th) 17 cells (116). *Bacteroides* genus was correlated with increased myeloid derived suppression cells (MDSCs) and the cytokines IL-8 and IL-13, which had roles in MDSC recruitment and proliferation, respectively (115). *Lactobacillus acidophilus* improved intestinal inflammation by modulating the balance of Th17 and Treg cells (117). The polysaccharide A from *Bacteroides fragilis* (118) facilitated the expansion and differentiation of intestinal Foxp3^+^ Tregs in addition to the production of IL-10 and TGF-β that regulate the functions of intestinal myeloid cells (119). Thus, the anti-inflammatory gut microbiota can potentially inhibit the formation of TLS in tumor environment.

### 6.2 Gut microbiota metabolites

Gut microbiota metabolites such as SCFAs, BA and Trp metabolites can inhibit inflammation. After interacting with GPR43 or GPR109A, SCFAs could regulate the activation of the NLRP3 and production of IL-18 to inhibit the activation of the Macs (120, 121). Butyrate also reprogramed Mac metabolism toward oxidative phosphorylation, causing an anti-inflammatory tolerant phenotype (122) through oxidative phosphorylation (OXPHOS) (123). Notably, SCFAs could down-regulate DC-secreted chemokines such as C-C chemokine ligand 5, up-regulate anti-inflammatory IL-10 and suppress DC maturation (124). SCFAs butyrate and propionate also inhibited LPS-mediated maturation of human monocyte-derived DCs *in vitro* (125, 126). SCFAs could promote differentiation and expansion of Treg cells. SCFA butyrate induced Treg cell differentiation through histone H3 acetylation at the Foxp3 promoter of the Foxp3 locus (127, 128), and fatty acid oxidation (129). Butyrate also supported DC-induced differentiation of Treg cells through butyrate receptor GPR109 and the butyrate transporter SLC5A8 in the DCs (130). Notably, the IL-10 expression in Th1, Th17 and Treg cells was promoted by SCFAs by suppression of histone deacetylases and regulation of the mTOR-S6K pathway (117). SCFAs also enhanced the generation of CXCR5^+^ T follicular helper cells *in vitro* and *in vivo*, which supported B cell differentiation (131). SCFAs, particularly butyrate stimulated ILCs via upregulating expression of AhR and HIF (132), the activation of the PI3K–AKT and mTOR signaling pathways in a GPR43-dependent manner to promote the proliferation of intestinal ILC1s and ILC3s (133). SCFA butyrate and propionate also regulated CD8 T cell activation via inhibiting IL-12 production in DCs. Taken together, SCFAs such as butyrate may inhibit TLS formation through immunosuppressive cells.

Through BA receptors TGR5, BA metabolites were essential to maintain tolerant phenotypes of the Macs (134–136). They modulated anti-inflammatory macrophage phenotype transformation and inhibited pro-inflammatory cytokine production through cyclic adenosine monophosphate (cAMP)-protein kinase A (PKA) pathway and regulating NLRP3 (137). The taurine-conjugated DCA (TLCA) also inhibited the expression of cytokines, such as IL-12 and TNFα and the expression of chemokines, CCL2, CCL 3, CCL4, CCL5, CXCL9, and CXCL10 in LPS-activated human Macs (138). DCA also inhibited the production of proinflammatory cytokines, such as IL-1β, IL-6, IL-12, IL-23, and TNF-α from LPS-activated DCs via TGR5 (139), whereas LCA suppressed the expression of proinflammatory cytokines via FXR (139). DC maturation and the inflammatory cytokine production were inhibited by VDR (140). DCA treatment also impeded Th1 and Th17 differentiation although LPS-activated DCs induced the differentiation of naïve CD4 T cells into Th1 or Th17 (141). BA derivatives isoalloLCA could selectively upregulate the expression of FoxP3 (142) through NR4A1, which could bind to 500 bp upstream of the *Foxp3* transcriptional start site (143, 144). It also promoted the differentiation of Tregs through mitochondrial reactive oxygen species (mitoROS) (142, 145, 146). 3-oxoLCA inhibited the differentiation of Th17 cells through RORγt (142). Notably, Foxp3 expression was also induced by BA metabolite isoDCA by reducing DC immune-stimulatory properties (145). Through increased transcription factors c-Maf and GATA-3, a shift from the Th1 to the Th2 phenotype was promoted by VDR activation (147). Through a VDR-dependent mechanism, the CD4 Th1 cell activation was suppressed by unconjugated BA metabolite LCA, causing diminished TNFα and IFNγ (148). BA metabolites also disrupted intracellular calcium homeostasis. The intracellular calcium was critical for NFAT (nuclear factor of activated T cells) signaling, which was necessary for T cells activation (149). The immune-metabolism in CD8 T cells could be reshaped by 24-Norursodeoxycholic acid (NorUDCA) to alleviate inflammation (150). Thus, these BA derivatives can also potentially inhibit the formation of TLS.

Trp metabolites from gut microbiota were essential in regulating the function of Macs via receptor AhR. Through suppressing histamine production, Trp metabolites could mediate suppression on inflammatory responses in the Macs (151). Trp metabolite 3-HAA inhibited LPS mediated NF-κB (nuclear factor κ gene binding) and PI3K (phosphatidylinositol 3 kinase)/Akt (protein kinase B)/mTOR (mammalian target of rapamycin) signaling pathways to reduce inflammatory cytokine production in the Macs (152). A recent report has demonstrated that the gut-bacterial-released indole and its metabolites (IPA and IAA) interacted with myeloperoxidase to inhibit its inflammatory function in the polymorphonuclear leukocytes at physiological concentrations (153). The kynurenine (Kyn), as an endogenous ligand of AhR, could induce AhR activation when generated in the tumor microenvironment (154), which promote tolerant phenotype in DCs to mediate the generation and expansion of Tregs.

Through AhR-ligand-Treg axis (155, 156), gut microbiota derived Trp metabolites promoted differentiation and function of Tregs. Kyn and its metabolites also enhanced Treg differentiation through the AhR (157–159), direct transactivation and the induction of epigenetic modifications, which controlled foxp3 transcription (160–162). 3-HAA in Kyn pathway also induced the differentiation and production of Treg cells via a nuclear coactivator 7 (NCOA7)-dependent pathway (163), and caused immune suppression by inducing apoptosis in T-cells through glutathione depletion (164). The expression of PD-1 in CD8 T cells, an immune inhibitory molecule, could be upregulated through Kyn (165). Through ROS pathway, the activity of NK cells was suppressed by Kyn, which could lead to cell death (166). Kyn also suppressed cytokine-mediated receptors, which were responsible for NK cells-mediated killing (167). Thus, Trp-indole derived metabolites also prevent the TLS formation through immunosuppressive cells.

### 6.3 Other anti-inflammation metabolites from gut microbiota

Other some gut microbiota derived metabolites also induce anti-inflammation responses, such as lactic acid (168), 12-ketolithocholic acid (169), linoleic acid (170), 5-hydroxyindoleacetic acid (171), polyamines (putrescine, spermidine, and spermine) (172), inosine (173) and hydrogen sulfide (174). Gut microbiota derived lactic acid induced transcriptional repression of macrophage inflammatory response via histone acetylation (168). It also served as a primary fuel source to promote histone H3K27 acetylation, which allowed the expression of immunosuppressive genes (168). Gut microbiota-derived 12-ketolithocholic acid could suppress the IL-17A secretion from colonic group ILC3 through down-regulated bile acid receptors, including vitamin D receptor (VDR) and pregnane X receptor (PXR) (169). The linoleic acid (LA) pathway in the gut microbiota determined the degree of inflammation and functions by suppressing Th17 differentiation and promoting Treg cell differentiation via the phosphorylation of Stat1 at Ser727 (170). Gut microbiota-derived 5-hydroxyindoleacetic acid alleviated colitis via MAPKs-PPARγ/NF-κB inhibition (171). The spermidine was produced by collective metabolic pathways of gut bacteria in immune cell regulation. It could facilitate polarization of Macs toward an anti-inflammatory phenotype, thus contributing to attenuation of inflammation (172). The inosine was a purine metabolite of *Akkermansia muciniphila* and *Bifidobacetrium pseudolongum* (77). The combination of *A. muciniphila* and inosine could modulate Treg cells and imbalance of Treg/Th17/Th1 cells through partly adenosine A2A receptor (173). The bacteria metabolized taurine or cysteine to form hydrogen sulfide (174). It could exert significant immune-regulatory roles by inhibiting the p38/ERK MAPK, p65 NF-κB, and JAK-STAT3 pathways and activating pathways such as Nrf2/HO-1, PI3K-AKT, and RAS/MAPK/AP-1 (175).

Taken together, besides inflammation associated gut microbiota and its structural components/metabolites, which can potentially promote the TLS formation, gut microbiota derived anti-inflammation metabolites such as SCFAs, BA and Trp metabolites, and other some metabolites also potentially inhibit the mature TLS formation through immunosuppressive cells in tumor environments. Notably, some metabolites derived gut microbiota such as SCFAs not only promote inflammation but also induce immunosuppression through different pathways.

### 6.4 Potential mechanisms of gut microbiota’s effects on TLS development

In immature TLS, besides T and B cells, there often exist immature and immune suppressive cells such as immunosuppressive Macs, myeloid-derived suppressive cells (MDSCs), immature DCs and Treg (26, 27). These immunosuppressive cells can potentially inhibit mature TLS formation. Follicular regulatory T cells (T_FR_ cells), characterized by the expression of CD25, FOXP3, glycoprotein A repetitions predominant and CXCR5, could suppress T_FH_ cells in B cell follicles with the involvement of TGF-β (176). DC–lysosome-associated membrane protein (DC-LAMP)^+^ mature DCs, which were potent antigen-presenting cells could form tight cell contacts with T cells, and were responsible for the activation of naive T cells and reactivation of T_CM_ cells (177). By contrast, T_reg_ cells found in this zone could disrupt the cross-talk, inhibiting the anti-tumor immune responses generated in TLS (111). Interestingly, following T_reg_ cell depletion, increased proliferation could be observed among CD4^+^ and CD8^+^ T cells within the TLS. T_reg_ cells present within TLS strongly expressed CTLA4 and CD39, suggesting that two inhibitory pathways were triggered by T_reg_ cells in TLS, through interaction with DCs and the production of adenosine, a potent inhibitor of T cells (111).

## 7 Effects of microbiota in tumor tissue on TLS formation

Besides gut microbiota metabolites, which can directly enter bloodstream to arrive in different tissues and organs, the microbes from the intestines can be transported via the bloodstream to tumor tissues through damaged blood vessels. Microorganisms include bacteria, viruses, fungus and others, among which viruses and bacteria have been found to be linked to the formation of TLSs (19, 178, 179). In tumor tissues, bacteria can release PAMPs (pathogen-associated molecular patterns) such as flagellins and LPSs to activate signaling pathways (MAPK, JAK–STAT, NF-κB) to release cytokines and chemokines, which promote the maturation of TLS. Typically, *H. hepaticus* colonization could induce *H. hepaticus*-specific T follicular helper (Tfh) cells, supporting the maturation of *H. hepaticus* ^+^ tumor-adjacent tertiary lymphoid structures in colorectal cancer (18). In hepatocellular carcinoma, the enrichment of the gut microbiota *Lachnoclostridium* was also associated with the presence of intratumoral TLS (19). Intra-tumor injection of *E. coli* MG1655 reprogramed tumor-associated macrophages into M1 phenotype that produce abundant CCL5, together facilitating tumor infiltration of adoptively transferred T cells. This effectively eradicated early-stage melanoma and inhibited the progression of pancreatic tumors (180). In addition, *Salmonella*-specific resident CX3CR1(hi) macrophages also induced tertiary lymphoid structures *in situ* (181). *E. coli strain Nissle 1917*, which could deliver cyclic di-AMP (a STING agonist) was shown to promote antigen presentation and type I interferon production by DCs, which were necessary for TLS formation (182). Innate immune cells activated by *S. Typhimurium* also released cytokines, such as IL-1β, TNF and IFNγ to induce inflammation, hereby transforming immune ‘cold’ tumors into ‘hot’ ones, which cause TLS (183).

## 8 Strategies of manipulating mature TLS formation

**Figure 6:**
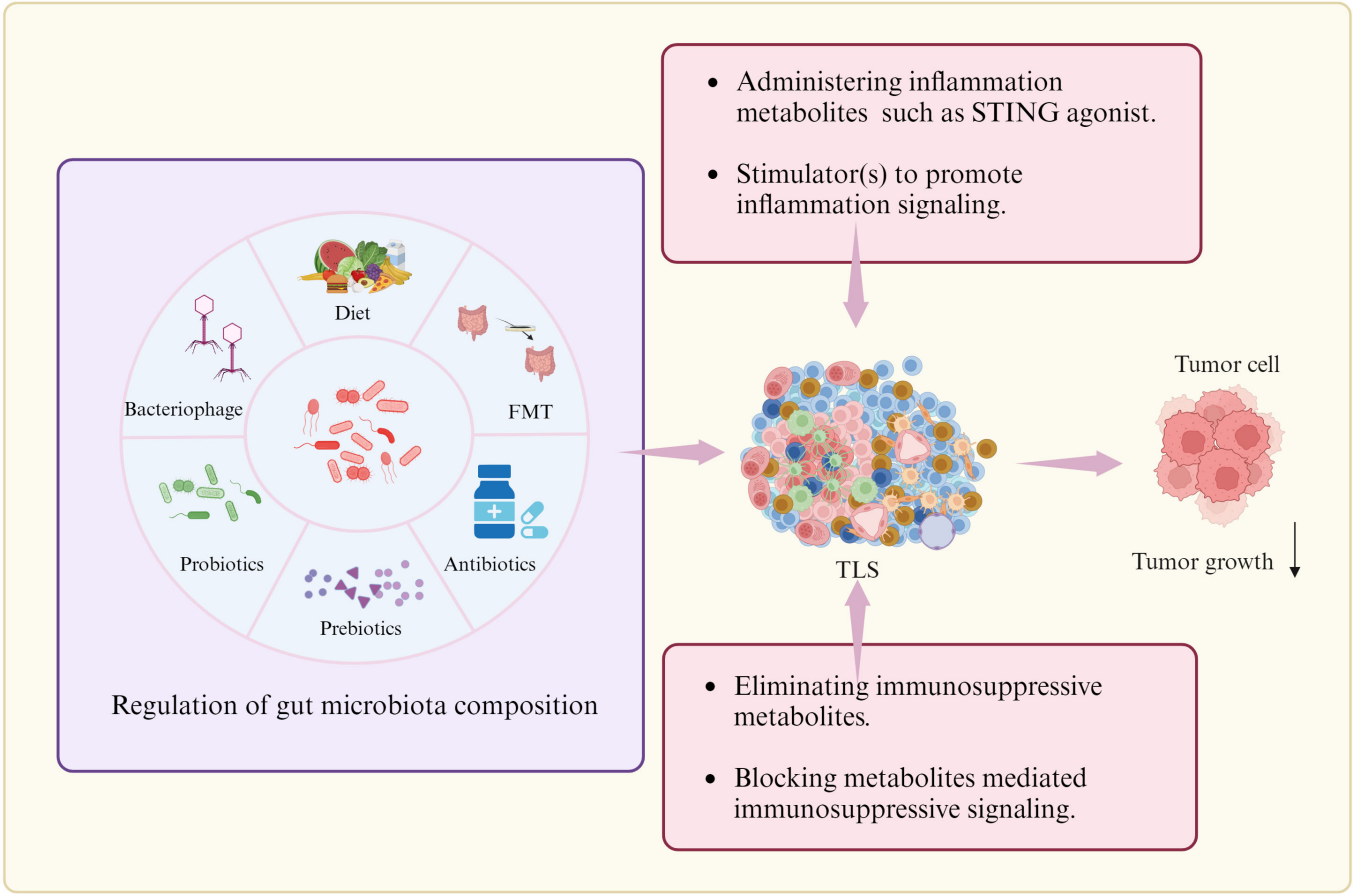
Potential strategies to regulate TLS formation. There are multiple strategies to promote TLS formation such as regulating the composition of gut microbiome through diet, FMT (Faecal microbiota transplantation), probiotics and prebiotics, and antibiotics or bacteriophages, administrating inflammation associated gut microbiota metabolites or stimulator to promote inflammation signaling, and eliminating immunosuppressive metabolites or blocking metabolites-mediated immunosuppressive signaling. Cells in TLS, see **Figure 1**.

Both antigen-specific antibody and T cell responses can be mounted in mature TLS. Various cancer treatments can also trigger TLS formation such as immunotherapy and cytokines (184). For instance, vaccination with human papillomavirus (HPV) oncoprotein vaccine could induce the formation of TLS (185). In pancreatic ductal adenocarcinoma (PDAC) mice, an anti-fibroblastic protein nanoparticle encoding LIGHT (Tumor necrosis factor superfamily member 14) induced intra-tumor TLS to inhibit abnormal collagen secretion (186). Low-dose radiotherapy could also trigger the development of immature TLS in a mouse model of lung cancer (184). Given the inextricable link between the gut microbiota and TLS, several potential strategies can be used to regulate mature TLS formation (**Figure 6**).

Regulation on gut microbiome composition. Regulation on the gut microbiome though diet, FMT (Faecal microbiota transplantation), probiotics and prebiotics, and antibiotics or bacteriophages can potentially improve the TLS formation. In murine models of CRC and melanoma, oral gavage with commensal *Clostridiales* strains could potently induce antitumor immunity through infiltration and activation of intratumoral CD8^+^ T cells. Accumulating studies have observed that the diversity and composition of host gut microbiota are associated with the efficacy of immunotherapy as well as the incidence of immune-related adverse events (irAEs). The commensal microbial community could also positively affect patient’s outcomes through activating CD8^+^ T cells-dependent antitumor response (187), enhancing antitumor T cell immunity by activating DCs via toll-like receptor 4 (TLR4) signaling in melanoma mice receiving radiation (188). Intact commensal bacteria were also found to support immune surveillance in mice with lung carcinoma partially via enhancing γδT17 cell response (189). Diet with fructooligosaccharides, the structural units of inulin fiber, can activate human Macs to produce pro-inflammatory cytokines (190). In mice, an insulin-based high-fiber diet upregulates microbiota-derived bile acid metabolites, which promote IL-33 production (191). Antibiotic therapy in animal studies has demonstrated its capacity to modify gut microbiota composition and associated metabolites (192). The next-generation probiotics, specifically *Eubacterium hallii*, *Faecalibacterium prausnitzii*, *Roseburia* spp., *Akkermansia muciniphila*, and *Bacteroides fragilis* can have an impact on the development of various diseases (193). Bacteriophages possess specific characteristics, including specificity for particular or closely related bacterial species. They can be as control agents in gut microbiota environments (194).

Administration of inflammation metabolite(s). Intra-tumoral administration of STING agonist ADU-S100 could promote TLS formation (92). STING agonist ADU S-100 -activated CD11c^+^ DCs also showed upregulated expression of TLS promoting factors *in vitro* and *in vivo* (92). cGAS-STING pathway initiated TLS formation through chemokine (101). Boosting Tfh cell numbers by initiating their differentiation, has also been proven as an efficient strategy to promote establishment of TLS in tumors.

Elimination of anti-inflammation metabolites. Immunosuppressive metabolites can be eliminated through multiple methods such as neutralization.

Blockage of immunosuppressive signaling(s). The activation or inhibition of immune-related signaling may cause immune cell aggregation and TLS formation, contributing to anti- tumor immunity (184). Elimination of immunosuppressive metabolites or blockage of metabolites-mediated immunosuppressive signaling should potentially improve the TLS formation. Repressing other regulatory cells such as macrophage subsets or B_reg_ cells may also favor formation of TLS.

## 9 Conclusion and perspectives

We here summarize the potential effects of gut microbiota/metabolites on the TLS formation, including inflammation-associated gut microbiota/metabolites, and also immune-suppressive gut microbiota/metabolites. Meanwhile, different strategies to promote the TLS formation also are suggested through regulating gut microbiota or gut microbiota associated pathways for anti-tumor immunity such as diet, FMT, probiotics and prebiotics, and antibiotics or bacteriophages, or administering inflammation metabolites or immunosuppressive metabolite inhibitor, or regulating inflammation or immunosuppressive mediated signaling. Notably, further studying the relationship between TLSs and microorganisms in cancer, especially the molecular network between them, will be helpful to fully understand the etiology and immune environment of cancer, and search for effective biomarkers and new treatments. Spatial transcriptomics can offer us a powerful tool to study the regulation of gut microbiota on TLS formation, which can capture the spatial distribution of RNA transcripts within the TME to phenotype all cell types and study their spatial organization within the tumor niche. Advancements in these technologies have paved the way for a deeper understanding of TLS, and hold great promise for precision medicine initiatives.
